# A Novel Fluorescent Biosensor for Adenosine Triphosphate Detection Based on a Metal–Organic Framework Coating Polydopamine Layer

**DOI:** 10.3390/ma11091616

**Published:** 2018-09-05

**Authors:** Peipei Xu, Guangfu Liao

**Affiliations:** 1 State Key Laboratory of Analytical Chemistry for Life Science, School of Chemistry and Chemical Engineering, Nanjing University, Nanjing 210093, China; 2 School of Materials Science and Engineering, PCFM Lab, Sun Yat-sen University, Guangzhou 510275, China; lgfhubu@163.com

**Keywords:** UiO-66, ATP detection, polydopamine layer, fluorescence assay

## Abstract

In this work, a novel and sensitive fluorescent biosensor based on polydopamine coated Zr-based metal–organic framework (PDA/UiO-66) is presented for adenosine triphosphate (ATP) detection. This PDA/UiO-66 nanoparticle which holds a great potential to be excellent fluorescence quencher can protect the 6-carboxyfluorescein (FAM)-labeled probe from cleaved by DNase I dispersed in solution and the flurescence of labeled FAM is quenched. When ATP molecules exist, aptamers on the PDA/UiO-66 nanoparticles can hybridize with ATP molecule to form complex structure that will be desorbed from the PDA/UiO-66 and digested by DNase I. After that, the released ATP molecule can react with another aptamer on the PDA/UiO-66 complexes, then restarts a new cycle. Herein, the excellent strong fluorescence quenching ability and uploading more amount of aptamer probes of PDA/UiO-66 composites make them efficient biosensors, leading to a high sensitivity with detection limit of 35 nM. Compared with ATP detection directly by UiO-66-based method, the LOD is about 5.7 times higher with PDA/UiO-66 nanoparticle. Moreover, the enhanced biocompatibility and bioactivity with PDA layer of the composites render a proposed strategy for clinical diagnosis field of detecting small biological molecules in vivo in the future.

## 1. Introduction

It is critically vital to develop a fast, sensitive, and cost-effective strategy for biomolecule detection in many fields, such as food safety and clinical diagnostic [1]. In recent decades, the fluorescent sensors based on photoinduced electron transfer (PET) mechanism or fluorescence resonance energy transfer (FRET) have received increasing attention due to their inherent advantage, e.g. convenient operation, high sensitivity, and high degree of automation [2]. The sensor commonly consists of a quencher and a fluorophore, of which the fluorescence change is coupled with the target recognition event. Until now, numerous fluorescence sensors based on nanomaterials—including graphene oxide, gold nanoparticles, and carbon nanotubes—have successfully constructed for biomolecules detection [3,4,5,6].

Multiply topologic metal–organic frameworks (MOFs) arouse great public concern over the years due to their natures of adjustable pore size [7,8,9]; high surface-to-volume ratio and robust thermal stability [10] and have been proved useful in areas as gas storage/separation; proton conducting; catalysis; sensing; drug delivery; and bio-imaging [11]. Considering the structures of MOF materials with abundant organic linkers containing a rich conjugated π-electron system [12], they also exhibit great affinities towards single-stranded DNA (ssDNA) through π-π stacking interactions. Furthermore, MOFs have great labeled fluorophores quenching ability with the mechanism of photoinduced electron transfer (PET) reaction [13] and can upload more probes ascribed to high surface areas, leading MOFs to a great potential application in fluorescence detection. Several fluorescence biosensors were presented based on MOFs. For example, Zhang’s group reported a Zr-based MOF/DNA hybrid system for Hg^2+^ detection [14]. Jiang’s group constructed an MOF as a sensing platform for DNA detection [15]. Dr. Ghosh’s group developed a selective and sensitive strategy to detect 2, 4, 6-Trinitrophenol (TNP) by a functionalized metal–organic framework [16]. Among all kind of MOFs, UiO-66, a Zr-based MOF, first reported by Lillerud et al., at 2008 [17], has caught attention of people recently due to its ease of synthesis and excellent chemical, mechanical, and thermal stabilities compared with other MOFs. 

Adenosine triphosphate (ATP) has an essential role to play in biological processes as it offers most of the chemical energy in living species [18,19]. It participates in various activities including muscle contraction, DNA replication, biosynthesis, enzyme catalysis, and neurotransmission in animate beings [20]. Another thing to note is that researchers find out different concentration of ATP is connected with common diseases like parkinsonism, Alzheimer’s diseases, hypoglycemia, and some malignant tumors [21]. Hence, the rapid and sensitive detection of ATP is of great important for pharmaceutical research and clinic treatment. Aptamers can form a hybridization structure after the nucleic acids specifically identifying targets and aptamers labeled with fluorophores have caught a large scale of attention [22]. They also have been developed as an intelligent fluorescent sensor for ATP detection. Among the aptamers-based strategies, polymers have been studied for the detection system and will continue to be researched in future. Thus, here we propose a system based on FAM-labeled aptamer probes quenched by UiO-66 nanospheres to fast detect ATP. However, unsaturated metal sites on the UiO-66 nanoparticle surfaces may give rise to UiO-66 nanoparticles conjugation with the phosphates on the end or side of the ssDNA and the Zr-phosphate interactions on the surfaces of the MOF nanoparticles may inevitably block the releasing of ssDNA from the surface of MOF nanoparticles [23].

**Scheme 1:**
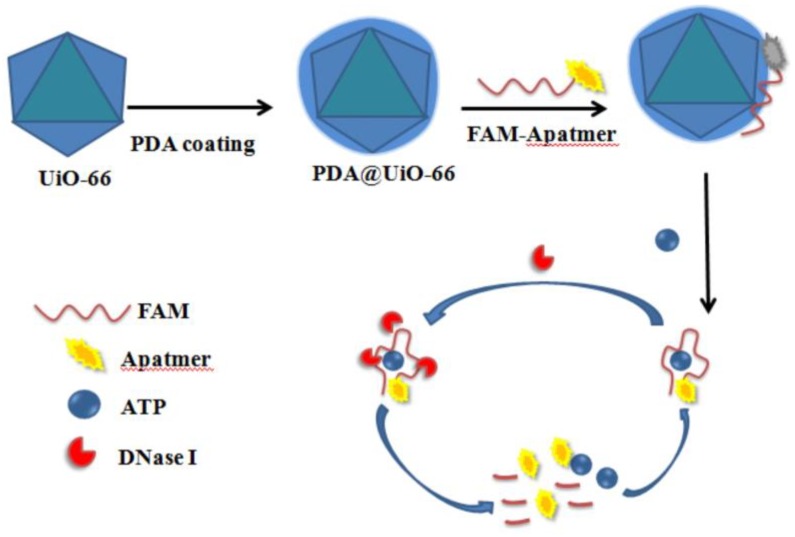
Schematic illustration of nuclease-assisted target recycling signal amplification strategy for ATP based on PDA/UiO-66 nanospheres.

It is reported that polydopamine (PDA) can independently functionalize surfaces to form adherent coatings with tunable thicknesses onto a wide range of materials including silica NPs, metal surfaces, and polymers [4,24]. It has also been demonstrated that PDA not only possesses affinities towards various ssDNA conformations [25] but also has a high fluorescence quenching effect through fluorescence resonance energy transfer (FRET). Functional groups of PDA, such as amino groups, are propitious to bring the ssDNA close to its surface in order to improve the quenching efficiency. It was a common material for plenty of in vivo studies attributed to the properties of high biocompatibility and bioactivity. As mentioned above, both MOF materials and PDA materials can be used for ATP detection, however, combined MOF materials with PDA materials for ATP detection is rarely reported. In addition, it is a promising material that can be used in vivo detection due to its high efficiency and biocompatibility resulted from the synergistic effect. Here, we have developed a novel PDA/UiO-66 complex as an efficient biosensor which can enhance quenching effect to reduce the background with synergistic effect between MOF and PDA. The synthesis of these nanoparticles does not change the MOF inherent advantageous properties of strong adsorption capacity, large surface area, and tunable size. An enzyme-assistant signal amplification strategy is induced to further improve the performance of biosensor, which relies on a single-labeled aptamer fluorescent probe adsorbed on PDA/UiO-66 complex and DNase I. DNase I is an endonuclease that can nonspecifically cleave DNA [26] while not active on DNA that adsorbed on PDA/UiO-66 complex [27]. Then the feasibility of employing PDA/UiO-66 complex and DNase I as a fluorescent sensing platform for target detection is confirmed. In the absence of ATP, single-stranded aptamers are adsorbed onto PDA/UiO-66 complex that preventing DNase I from suffering the cleavage of the constrained aptamers. However, when reacted with ATP, the ATP/aptamer hybridization structures bind weakly to PDA/UiO-66 and then desorb from the surface of PDA/UiO-66. Without the protection of PDA/UiO-66, aptamers are immediately hydrolyzed by DNase I. After digesting, the released ATP will hybridize another aptamer on the PDA/UiO-66 surface and restart a new cycle. Herein, the released fluorophores generate strong fluorescence intensity. The concentration of ATP can be detected by monitoring the fluorescence change. The remarkable fluorescence quenching ability of PDA/UiO-66 complexes makes them efficient biosensors, leading to a high sensitivity with detection limit of 35 nM. This LOD is about 5.7 times higher than that of the UiO-66-based method without PDA modification. The working principle based on the PDA/UiO-66 platform for the assay of ATP was illustrated in Scheme 1.

## 2. Experimental

### 2.1. Materials 

The FAM-labeled Adenosine triphosphate (ATP) aptamer: 5′-FAM-ACCTGGGGGAGTATTGCGGAGGAGGAAGGT-3′ was synthesized by Shanghai Sangon Biotechnology Co., Ltd. (Shanghai, China). Adenosine triphosphate (ATP), uridine triphosphate (UTP), cytosine triphosphate (CTP), guanidine triphosphate (GTP), and DNase I were also all bought from Shanghai Sangon Biotechnology Co., Ltd. (Shanghai, China). Tris-HCl buffer (pH 7.4) containing 100 mM NaCl and 2 mM MgCl_2_ was prepared with concentration of 20 mM. DNA solution was dissolved into 20 mM Tris-HCl buffer mentioned above. ZrOCl_4_, 1,4-benzenedicarboxylic acid, acetic acid, dopamine hydrochlorideand, *N*,*N*-dimethylformamide (DMF), ethanol were all purchased from Sigma-Aldrich (St. Louis, MO, USA). All the materials and solvents were reagent grade and all solutions were prepared with the water from a Milli-Q system (Milli-pore, New York, NY, USA). Transmission electron microscopy images were recorded on a Model JEM 2100 high-resolution TEM microscope (JEOL, Tokyo, Japan). Scanning electron microscopy (SEM) images were recorded with a Hitachi Model S-4800 SEM microscope (Tokyo, Japan). Infrared microscopy images were obtained on a Nicolet NEXUS870 transform infrared spectrometer (Madison, WI, USA). X-ray diffraction (XRD) data were collected on a Bruker Advance D8 diffractometer (Bruker, Germany) at 40 kV, 40 mA for Kα (λ = 1.5418 Å) with a step size of 0.02° in 2θ ranging of 5–50°. The fluorescence excitation and emission spectra were recorded with a Hitachi F-7000 FL spectrophotometer (Tokyo, Japan) equipped with a 150 W Xenon lamp as an excitation source at room temperature.

### 2.2. Preparation of UiO-66

The UiO-66 nanoparticles were synthesized on the basis of the published approach [28]. In simple terms, 315mg ZrOCl_4_ (0.09 mM) and 598 mg 1,4-benzenedicarboxylic acid (0.24 mM) were mixed and dissolved in 60 mL of DMF in a 100 mL reagent bottle. Then, 30 mL acetic acid was added into the mixture. After that, the mixed precursor solution was heated in oven with a temperature of 120 °C for 48 h. We could observe the production of precipitation that was the anticipated UiO-66 nanoparticles. After cooling down to room temperature, the outcome was washed with DMF and ethanol for three times respectively and collected by centrifuging at 8000 rpm for 5 min. Finally, UiO-66 nanoparticles were dried under vacuum at ambient temperature for further use.

### 2.3. Preparation of PDA/UiO-66

Dopamine hydrochloride (4 mg) was dissolved in a mixed solvent of Tris-HCl buffer (20 mL, pH 8.5) containing 40 mg UiO-66, then the solution was stirred at room temperature for 16 h. After modification, the PDA/UiO-66 was obtained by centrifugal removal of the entrapped template molecules and then purification by washing with Tris-HCl buffer (pH 7.4) followed by drying under vacuum at ambient temperature. It was under drying preservation at room temperature before use.

### 2.4. Fluorescence Measurements

5 mg PDA/UiO-66 sample was ultrasonically dissolved into 1 mL Tris-HCl buffer (pH 7.4). A concentration of 1 µM the FAM-labeled ATP-aptamer solution was obtained by dilution of the stock solution in Tris-HCl buffer. Following that 40 µL of above ssDNA aptamer and an aliquot of PDA/UiO-66 suspension liquid were in sequence added into Tris-HCl buffer and incubated for 10 min. Afterwards, DNase I and different concentrations of ATP solution were mixed in Tris-HCl buffer and reacted at room temperature. Finally, the fluorescence intensities were recorded. To contrast with PDA/UiO-66, UiO-66 was also introduced in the system above discretely.

## 3. Results and Discussion

The UiO-66 nanosphere was produced by mixing ZrOCl_4_ and 1,4-benzenedicarboxylic acid in DMF solution with a simple one-pot solvothermal method. Acetic acid was added into the mixture before the reaction happened to modulate the size of well-defined octahedral shape monodisperse UiO-66 crystals. After that, the UiO-66 was coated on a PDA layer in a PH 8.5 dopamine solution at room temperature.

**Figure 1:**
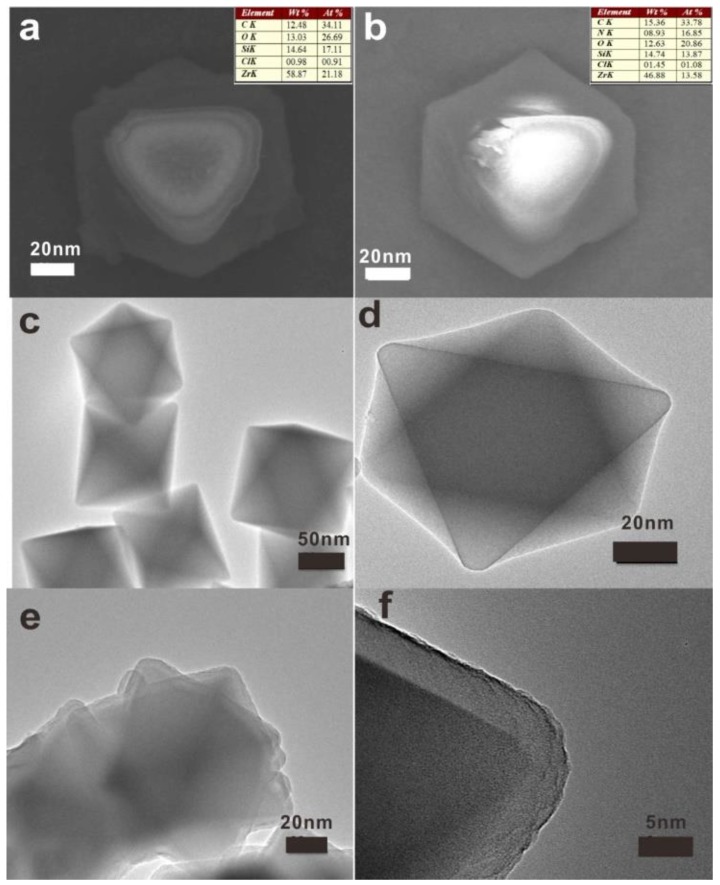
SEM image of the as-prepared UiO-66 (**a**) and PDA/UiO-66 nanocomposites (**b**); the insets represents the element content of UiO-66 and PDA/UiO-66 nanocomposites, respectively, tested by EDX. TEM images of UiO-66 (**c**,**d**) and PDA/UiO-66 nanocomposites (**e**,**f**).

**Figure 2:**
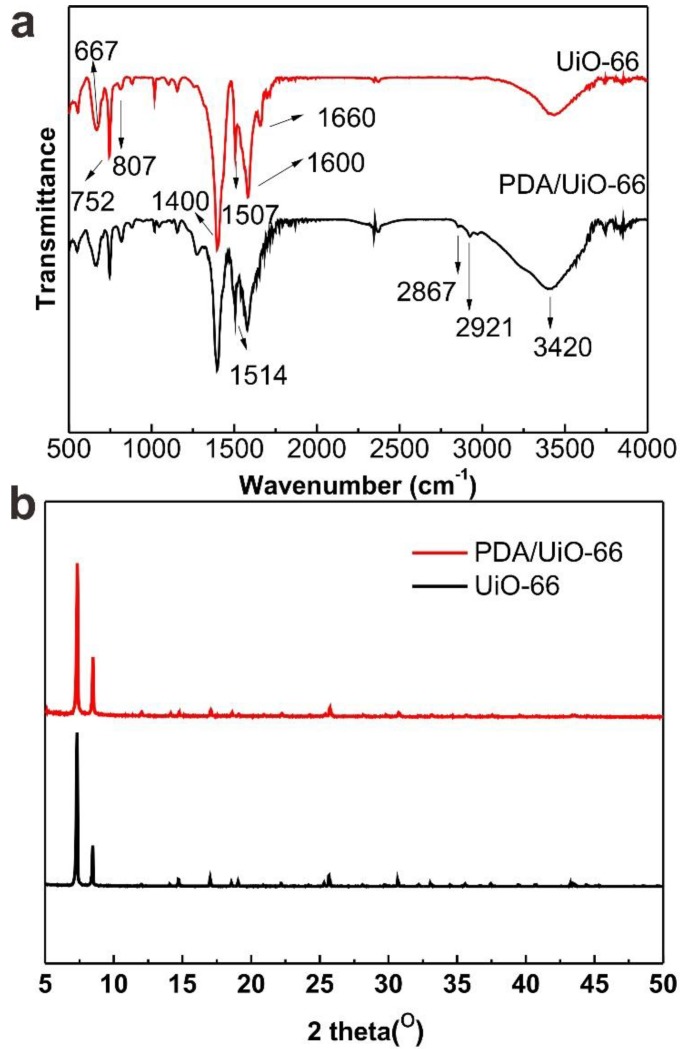
XRD pattern profiles (**a**) and IR spectrum (**b**) of UiO-66 and PDA/UiO-66 nanocomposites.

The UiO-66 crystalline is a coordination of hexaunuclear Zr oxohydroxo clusters and organic aromatic linker topological structure. The diameter of expected UiO-66 was determined about 110 ± 10 nm from the scanning electron microscopy (SEM) and transmission electron microscopy (TEM) images (Figure 1a,c,d) which were recorded to characterize the crystallite size and morphology. As was shown in Figure 1, SEM (Figure 1a) and TEM images (Figure 1c,d) revealed that the reaction yielded monodisperse crystals with an average size of 110 nm and all crystals exhibit a definite octahedral shape. As mentioned above, dopamine can autopolymerize to form a thin polydopamine layer for solid surface modification under alkaline condition. After a stable polydopamine layer self-assembly imprinted on the UiO-66 nanosphere, TEM images showed that the as-prepared PDA/UiO-66 nanocomposite synthesized with the ratio of UiO-66 to dopamine about 10:1 was worked out (Figure 1d) and corresponding to the polydopamine shell thickness of ~4 nm indicated by the aggregate (Figure 1f). Moreover, after PDA was composited with UiO66, the N element content was increased and Zr, Cl, and O element content was decreased, this indicated the PDA was successfully composited with UiO66. These results suggested that both nanoparticles were successfully prepared and the morphology was not significantly changed after self-assembly coating. Besides, the UiO-66 and PDA/UiO-66 structures could also be confirmed by infrared spectroscopy (IR) and X-ray diffraction (XRD). The IR spectra of UiO-66 and PDA/UiO-66 samples were presented in Figure 2a. The IR peaks detected at 667, 752, and 807 cm^−1^ were likely associated with OH and C–H vibrations originated from the benzenedicarboxylate ligand while the infrared absorption spectrum detected at 1507 cm^−1^ was attributed to the aromatic rings vibration. The IR bands positioned at 1400 and 1600 cm^−1^ respectively were due to the typical O–C–O asymmetric and symmetric stretching of benzenedicarboxylate ligand. Besides, the small spectral band located at 1660 cm^−1^ represented the stretching vibrations of C=O of the carboxylic acid in benzenedicarboxylate [29,30]. After reaction with dopamine, we could see that a weak peak appeared at 1514 cm^−1^ which was attributed to the bending vibration of -NH group. The characteristic absorption peak at 2867 and 2921 cm^−1^ could be assigned to the –CH_2_ group from PDA and peak at 3420 cm^−1^ was enhanced by stretching vibration of aromatic –OH and –NH. All evidence indicated that PDA layer coating exactly onto the UiO-66 nanoparticles. In order to confirm the synthetic universality of this protocol, structure stability of the UiO-66 framework was further indicated by XRD investigation. For the UiO-66, We could observe two typical strong diffraction peaks at 2θ of 7.4°, 8.5° assigned to (111), (002) crystallographic planes respectively along with 12.1°, 14.2°, 17.1°, 22.3°, 25.7°, 31.1°, and 33.1°, which were indexed as the reflections of (022), (113), (004), (115), (224), (046), and (137) crystallographic planes of UiO-66 and they were all well suited to the literature [31]. The data highly proved the formation of the octahedral crystalline structure. In the case of PDA/UiO-66, the XRD patterns displayed almost no difference with the pristine UiO-66 although the XRD diffraction peaks exhibited little enhance in the intensity. This result demonstrated that the primary crystallite structures of UiO-66 MOFs were well resolved and remained nearly unchangeable after the reaction.

**Figure 3:**
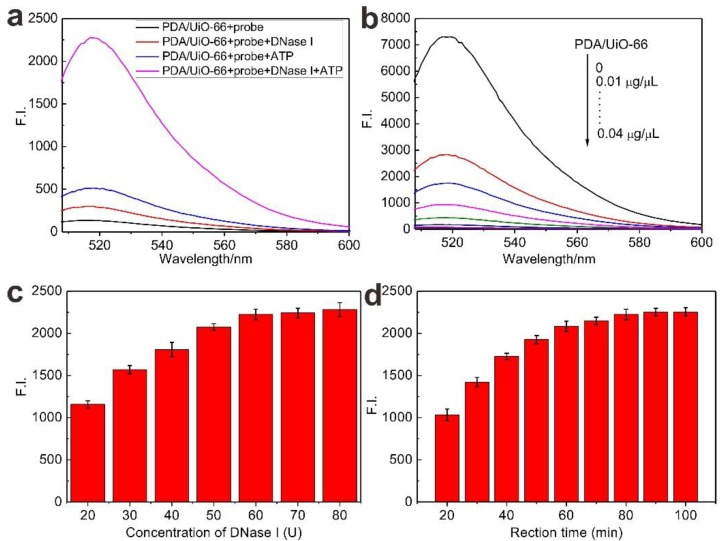
Effects of (**a**) fluorescence emission spectra of the probe (100 nM) under different conditions; (**b**) fluorescence emission spectra of probes with different concentration of PDA/UiO-66 (0.01, 0.015, 0.02, 0.025, 0.03, 0.035 and 0.04 µg/µL); (**c**) immobilization time for aptamer complex reacted with 100 µM ATP and amplified by cleavage of DNase I; (**d**) fluorescence intensity of different the concentration of DNase I applied in the platform. The data points and error bars represent average and standard deviations of measurements at different times (n = 3 for error bars).

As we all know, the selected aptamers can specifically recognize different targets ranging from small organic or inorganic molecules to macromolecules or even cells. In this work, modified aptamer probes underwent a distinct conformational change that can be digested under the catalyzing of DNase I when challenged with ATP, ultimately releasing the ATP. FAM got far away from the nanosphere surface and was dissociated free in the solution, and then the fluorescence intensity recovered again. Strategy of DNase I-assisted signal recycling amplification of platform was commonly utilized in analytical assays. Fluorescence measurements were operated to confirm the recycling process and signal recovery. Dramatic reduction of the fluorescence intensity was observed in Figure 3a after probes incubation with PDA/UiO-66 which indicated high adsorption and quenching ability efficiency of the nanomaterials (black line). Upon the addition of the ATP, ssDNA probes on PDA/UiO-66 surfaces hybridized with ATP to form complex structures and removed from nanospheres which quenched FAM fluorescence. While without ATP, only DNase I could also cause a slight fluorescence change probably due to a number of probes hydrolyzed by the enzymes, but most probes were protected well from DNase I digestion (red line). When targets existed, there was an increase of the fluorescence intensity (blue line). In contrast to the presence of both DNase I and targets, a remarkable fluorescence enhancement was achieved (pink line). This result fully demonstrated the success of the ssDNA probe cleaved by DNase I catalysis, releasing the targets to absorb another ssDNA probe from PDA/UiO-66, and a digesting reaction that led to an effective amplification. 

Detection of ATP hinged on a recycling enzymatic reaction triggered by DNase I digesting ssDNA probe desorbed from novel PDA/UiO-66 nanospheres after target-induced hybridization to form a rigid structure and liberating ATP to act with ssDNA probe again. Fluorophores labeled on the digested probes were free into solution and caused the fluorescence recovery. To obtain the best analytical performance, the concentration of PDA/UiO-66 applied for the platform to quench fluorescence was optimized. As seen in Figure 3b, the impact of PDA/UiO-66 was studied at the concentration from 0 to 0.04 µg/µL. Along with the incremental concentration of PDA/UiO-66, the fluorescence of FAM in the system rapidly decreased at beginning then slowed down the rate and was almost entirely quenched in presence of 0.03 µg/µL PDA/UiO-66 with the quenching efficiency of 99.1%.

**Figure 4:**
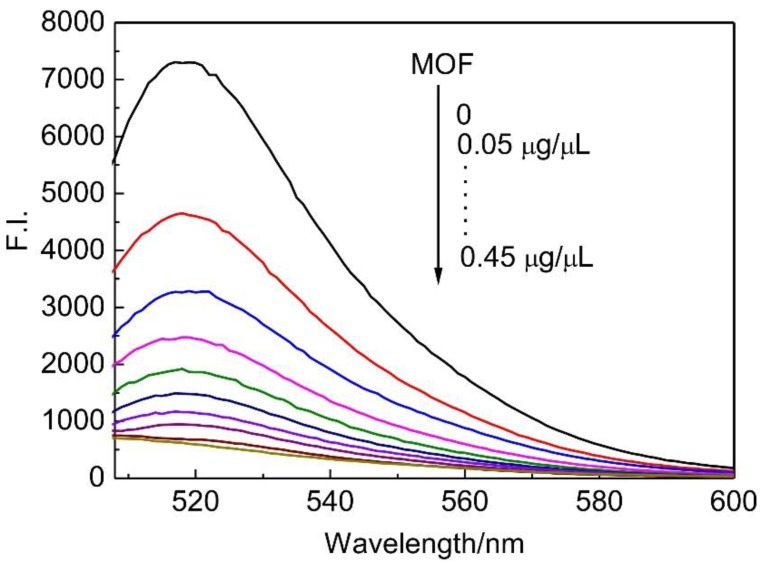
Effects of fluorescence emission spectra of probes with different concentration of UiO-66(0, 0.05, 0.10, 0.15, 0.20, 0.25, 0.30, 0.35, 0.40 and 0.45 µg/µL).

Compared with ssDNA/UiO-66 system (Figure 4), the fluorescence decreased the same way with increasing concentration of UiO-66 and got the largest quenching efficiency of 91.6% at last at a concentration of 0.45 µg/µL. The optimized concentration of PDA/UiO-66 for the experiment was 0.03 µg/µL as a consequence. For the enzyme-based amplification, the amount of DNase I was also investigated to improve the property of resulting biosensor (Figure 3c). The result clearly showed that the signal increased with the increasing concentration of DNase I and reached a plateau at 60 U, which was determined as experimental amount. In addition, the incubation time for recycling reaction also had an important influence and was studied ranging from 20 min to 100 min in this work (Figure 3d). The fluorescence recovery almost achieved a balance value up to 80 min, reflecting that 80 min could be enough time for the experiment.

**Figure 5:**
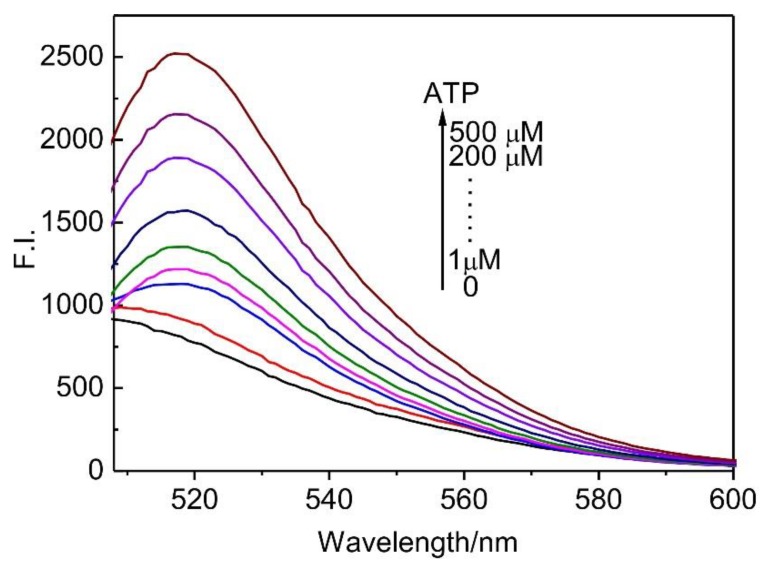
Effects of fluorescence emission spectra of probes with different concentration of ATP (0, 1, 5, 10, 20, 50, 100, 200 and 500 µM).

**Figure 6:**
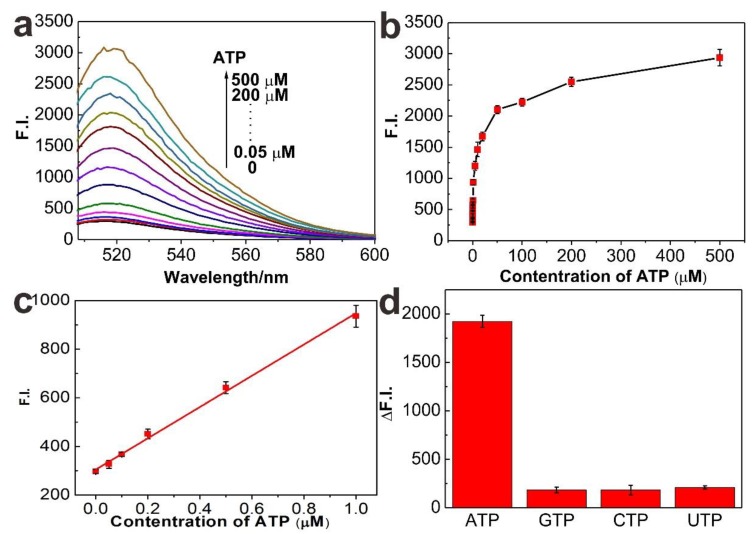
(**a**) Fluorescence emission spectra of the system with DNase I in the presence of different concentration of ATP (0, 0.05, 0.1, 0.2, 0.5, 1, 5, 10, 20, 50, 100, 200 and 500 µM); (**b**) calibration curve for ATP detection from 0–500 µM; (**c**) amplification of the concentration from 0–1 µM with standard curve equation: *A* = 644.830 [*C*] + 306.428 (*R*^2^ = 0.9969); (**d**) fluorescence intensity recovering after the sensing platform were challenged with 100 µM ATP, GTP, CTP, and UTP respectively with 60 U DNase I incubated for 80 min. Signal recover values for FAM were all less than 3% after introduction of the analogs compared with ATP resulting in 26.3 %.

UiO-66 was also introduced in the system above discretely (Figure 5). Under optimum conditions above, different concentration of ATP from 0 to 500 µM were added to the system and could be detected by recording the corresponding fluoresence intensity at 518 nm recovered with the increasing ATP concentration proportionally and gradually ended to a plateau (Figure 6a,b). We found out that the fluoresence intensity indicated a linear relationship over a low ATP concentration range from 0 to 1 µM (Figure 6c). The detection limit (LOD) for ATP calculated to be 35 nM at 3σ was about 5.7 times higher than that of method based on UiO-66 with LOD of 200 nM. This consequence was probably attributed to the unsaturated Zr metal sites on the UiO-66 nanoparticle surface conjugation with ssDNA probes through phosphate-Zr bonds which blocked the releasing of ssDNA from the surface of nanoparticles. The coating PDA layer effectually avoided the possibility of the coordination and improved detection sensitivity. Furthermore, this strategy achieved much lower detection limit than the biosensor based on the graphene oxide with the LOD of 10 µM and was also of high sensitivity in contrast to the polydopamine nanospheres intergrating with Exo III-assisted target recycling developed as 0.2 µM (Table 1). These results demonstrated that the strategy with DNase I-assisted recycle amplification formation could be used for efficient and sensitive detection of ATP with corresponding signal readouts. Three analogs—GTP, CTP, and UTP—were introduced to the system as interferent targets to determine the aptamer-based platform only in response to ATP by comparison with the absorbance of the working solutions. Figure 6d displayed the fluorescence intensity slightly changes below 3% with 100 µM analogs respectively but probably 26.3% signal change in the presence of same concentration of ATP, indicating the high sensitivity of the platform.

## 4. Conclusions

In conclusion, we proposed a very simple method for the preparation of novel PDA/UiO-66 composites. This new synthesized PDA/UiO-66 nanomaterial offered a straightforward, fast, and simplified fluorescence strategy with signal turn-on state for the detection of ATP. PDA coating avoided the phosphates of oligonucleotides coordination with unsaturated metal sites Zr on the MOF nanoparticle surfaces which also improved the sensitivity of this system to accurately recognize targets according to the experiment. Moreover, synergistic effect between MOF structure and the PDA layer could enhance the quenching performance of the nanomaterials. The signal produced by this biological assay was significantly amplified with the assistance of DNase I, which could largely improve the reaction effectiveness and led to an increase of the signals. We believe that this kind of fluorescence biosensor based on PDA/UiO-66 with higher biocompatibility and bioactivity could potentially be utilized for cellular analysis and be applied in subsequent analysis in the near future.

## Figures and Tables

**Table 1:** Comparison of lower limit of detection for various materials.

Probe	Detection Method	Lower Limit of Detection	Reference
FAM-labeled aptamer	Fluorescent detection using PDA/UiO-66 nanoparticles	0.035 μM	This work
FAM-labeled aptamer	Fluorescent detection using UiO-66 nanoparticles	0.2 μM	This work
Graphene oxid-based multicolor fluorescent DNA nanoprobe	The biosensor based on the graphene oxide	10 μM	[32]
Thiolated aptamer	A sandwich dipstick assay based on split aptamer fragments	0.5 μM	[33]
Nuclease-assisted label-free aptamer	Fluorescent detection using copper nanoparticles	0.093 μM	[34]
Noncovalent assembly of carbon nanoparticles and aptamer	Assay based on carbon nanoparticles	0.1 μM	[35]

## Abbreviations/Nomenclature

PET	photoinduced electron transfer
FRET	fluorescence resonance energy transfer
MOFs	metal-organic frameworks
ssDNA	single-stranded DNA
TNP	2,4,6-trinitrophenol
ATP	adenosine triphosphate
PDA	polydopamine
LOD	detection limit
UTP	uridine triphosphate
CTP	cytosine triphosphate
GTP	guanidine triphosphate
